# Supplementary figures and images for: Unveiling the intercompartmental signaling axis: Mitochondrial to ER Stress Response (MERSR) and its impact on proteostasis
Source: PLoS Genet. 2025 May 8;21(5):e1011700. doi: 10.1371/journal.pgen.1011700 (PMC12088515; doi:10.1371/journal.pgen.1011700)

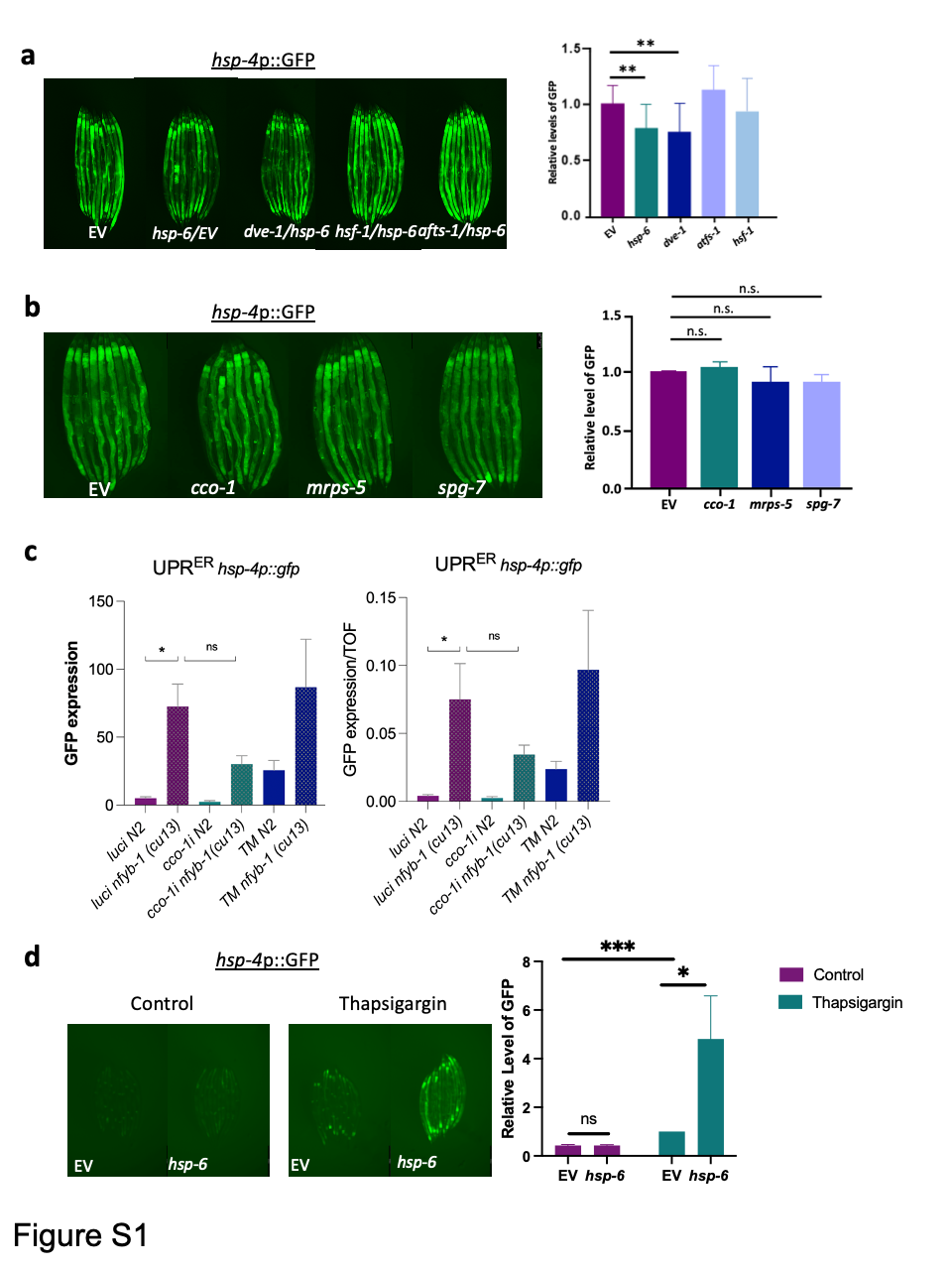

Supplement: S1 Fig — a) Inhibition of UPRER by hsp-6 RNAi is regulated through the dve-1 transcription factor. Animals were treated with tunicamycin as described above for 4 hours on day 1 of adulthood, followed by transfer onto RNAi plates, which targeted specific transcription factors within the mitochondria or cytosolic stress pathway. The animals were imaged at day 3 adult. b) Post-development mitochondrial stress through knockdown of different mitochondrial proteins has a different effect on the induction of UPRER. Animals were treated with tunicamycin at L4, then were treated with the indicated RNAi or the empty vector control. Animals were imaged at day 3 adult. Graph shows mean+/-SD of four biological repeats, n>=8. c) Developmental mitochondrial stress by cco-1 knockdown also does not affect UPRER. UPRER (hsp-4p::GFP) reporter strains in wild-type (N2) or nfyb-1 (cu13) background were treated with control (luciferase) or cco-1i RNA from hatch, and GFP expression was measured on day 1 of adulthood with biosorter (left), and the intensity was normalized with the size of the worms (TOF: time of flight). Statistics determined by one-way ANOVA, ns: not significant, * p<0.5, ** p<0.01, Error bar shows mean± s.e.m. d) UPRER induced by Thapsigargin treatment is not suppressed by MERSR. The graph shows the mean+/-SD of the images of 10-20 animals (three biological replicates). Each RNAi-treated cohort was compared to the Thapsigargin-treated EV control to assess GFP induction. Mock-treated controls are shown on the left, indicating the basal expression of GFP. (TIFF) [file pgen.1011700.s001.tiff]

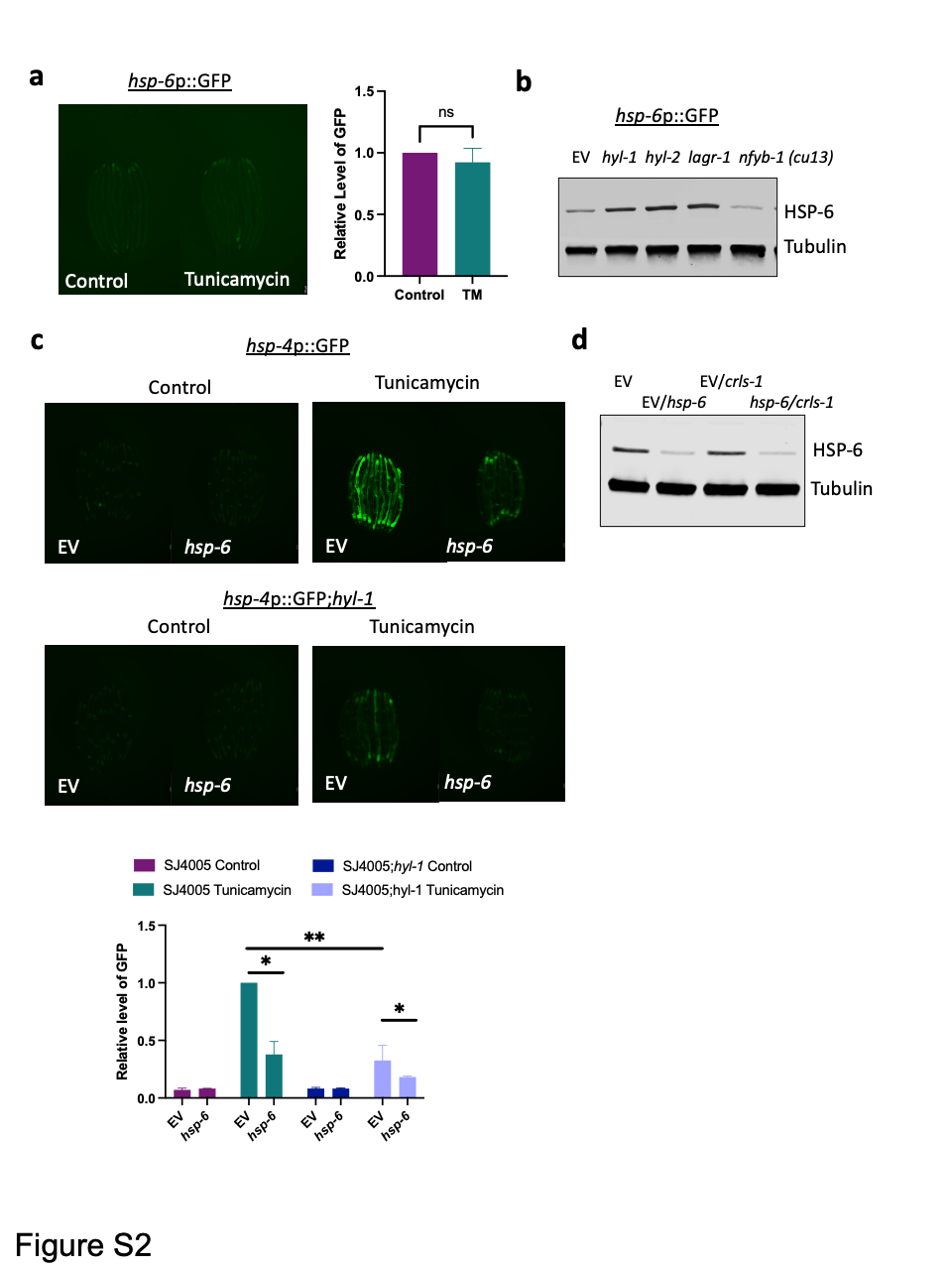

Supplement: S2 Fig — a) Tunicamycin treatment does not induce UPRMT. hsp-6 reporter animals (SJ4100) were treated with tunicamycin as described above. The animals were imaged on day 3. The animals were imaged on day 3. b) HSP-6 levels following indicated treatment, including RNAi of hyl-1, hyl-2, and lagr-1 or hsp-6 reporter animals crossed with nyfb-1 mutant (hsp-6p::GFP;nyfb-1). Animals were treated with indicated RNAi on day 1 adult and imaged on day 3. c) The hyl-1 deletion mutant with UPRER reporter background. The UPRER (hsp-4p::GFP) reporter strain (SJ4005) is crossed with hyl-1 (ok976) deletion mutant strain to measure UPRER activation following tunicamycin treatment. hyl-1 (ok976) animals were treated with tunicamycin and RNAi as described above. The graphs show the mean + /-SD of the images of 10–20 animals (three biological replicates). Each RNAi-treated cohort was compared to the tunicamycin-treated EV control to assess GFP induction. Mock-treated controls are shown on the left, indicating the basal expression of GFP. d) Western blotting of HSP-6 after double RNAi treatment in Fig 2C. HSP-6 levels were comparable between EV/hsp-6 double RNAi and crls-1/hsp-6 double RNAi. (TIFF) [file pgen.1011700.s002.tiff]

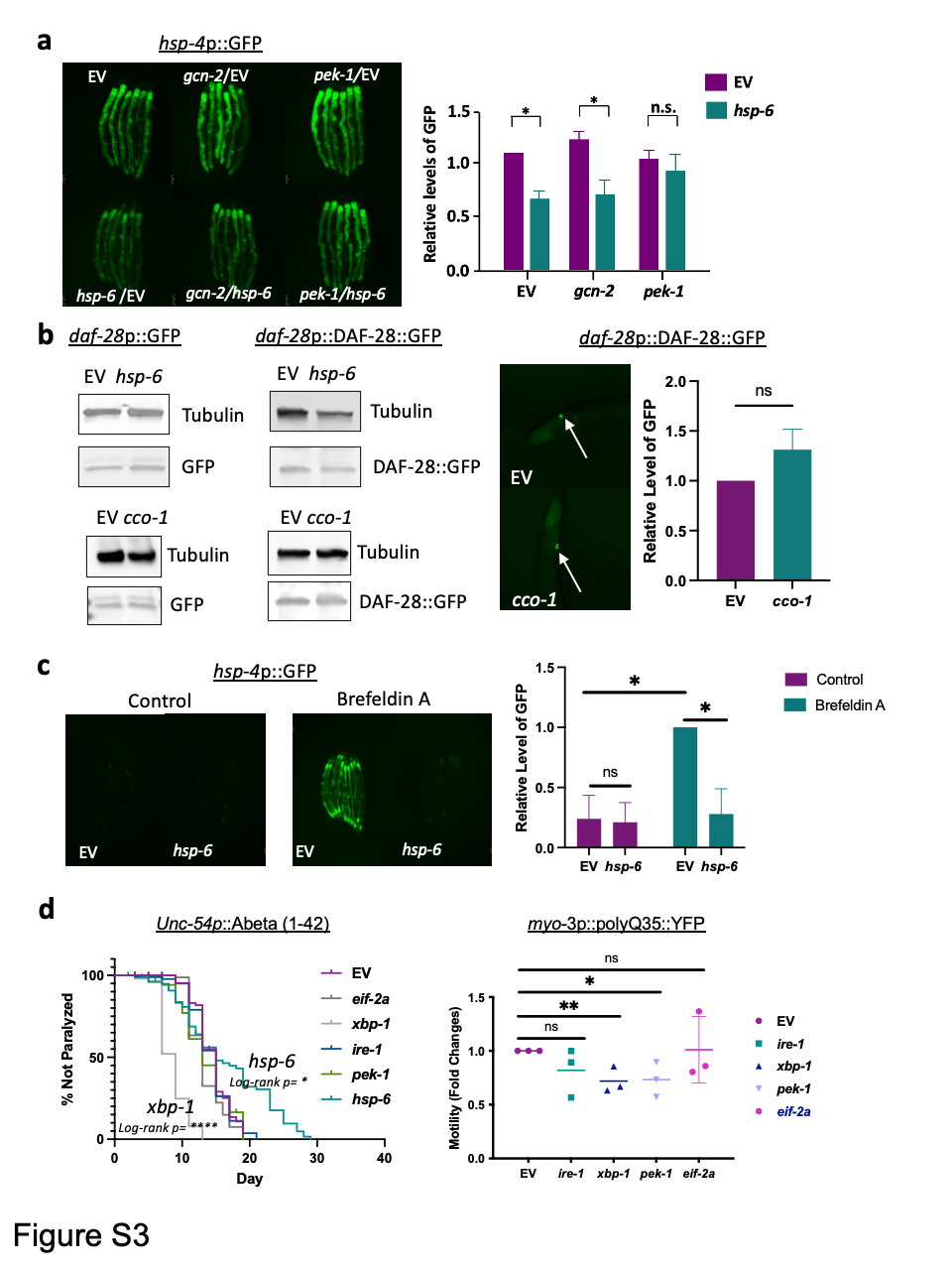

Supplement: S3 Fig — a) pek-1 mediates the suppression of UPRER. Animals were treated with tunicamycin and the indicated RNAi as previously described (Fig 1). Graph shows mean + /-SD of three biological repeats, n>=6. b) (left) Western blot of daf-28p::GFP and DAF-28::GFP shows that hsp-6, ire-1, xbp-1 or cco-1 RNAi treatment does not change daf-28 transcriptional and translational expression levels, suggesting that the reduction of daf-28 exhibited in the coelomocytes in Fig 3F is most likely the result of a decrease in ER secretory function. (right) DAF-28::GFP secretion to coelomocytes were unchanged with UPRMT induced by cco-1 RNAi. The graph shows mean + /-SD of GFP intensity normalized to empty vector control, n>=10 with three biological repeats. c) UPRER induced by Brefeldin A treatment is also suppressed by MERSR. The graph shows the mean + /-SD of the images of 10–20 animals (three biological replicates). Each RNAi-treated cohort was compared to the Brefeldin A-treated EV control to assess GFP induction. Mock-treated controls are shown on the left, indicating the basal expression of GFP. d) Proteotoxicity models expressing Abeta (1–42) or polyglutamine (Q35) within body wall muscles. Individual RNAi of UPRER components followed by paralysis assay (left) and motility assay (right). (TIFF) [file pgen.1011700.s003.tiff]

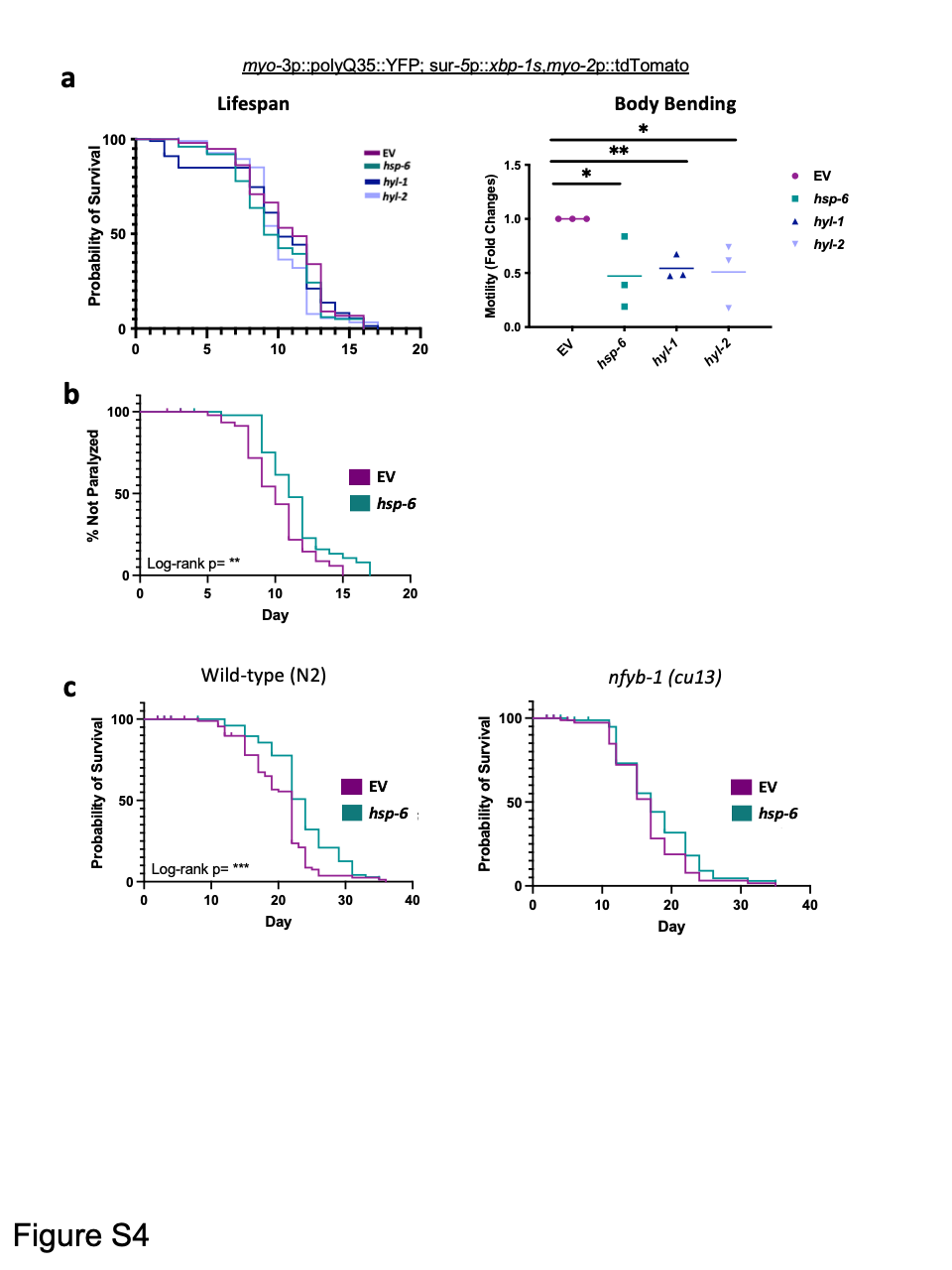

Supplement: S4 Fig — a) (left) Lifespan of the animals expressing polyQ35::YFP in their body wall muscle (AM140) with constitutively active UPRER in the whole-tissue by expressing xbp-1s (myo-3p::polyQ35::YFP; sur-5p::xbp-1s,myo-2p::tdTomato). Log-rank * p < 0.05 for EV vs hyl-2. (right) Motility was determined using a body bending assay that measures the number of body bending per 30 seconds. The relative motility was plotted by normalizing with empty vector control, and the rate was compared to that of the empty vector control (Three biological repeats with n > 10, mean + /- SD). Statistics determined by t-test comparing each condition to EV control, * p < 0.5, ** p < 0.01. b) Paralysis assay of GMC101 animals that express Aβ (1–42) in their body wall muscle. The assay was performed at 25°C as previously described [73]. hsp-6 was knocked down from day 1 of adulthood. c) (left) Lifespan of wild-type N2 worms treated with hsp-6 RNAi. Animals were transferred to RNAi-containing plates on day 1 adult. P < 0.001. (right) Lifespan nfyb-1(cu13) mutant animals following hsp-6 knockdown from day 1 of adulthood. hsp-6 RNAi moderately extended the lifespan (Log-rank ****p < 0.0001) of wild-type animals, whereas it did not significantly affect the lifespan of nfyb-1 mutant animals. (TIFF) [file pgen.1011700.s004.tiff]

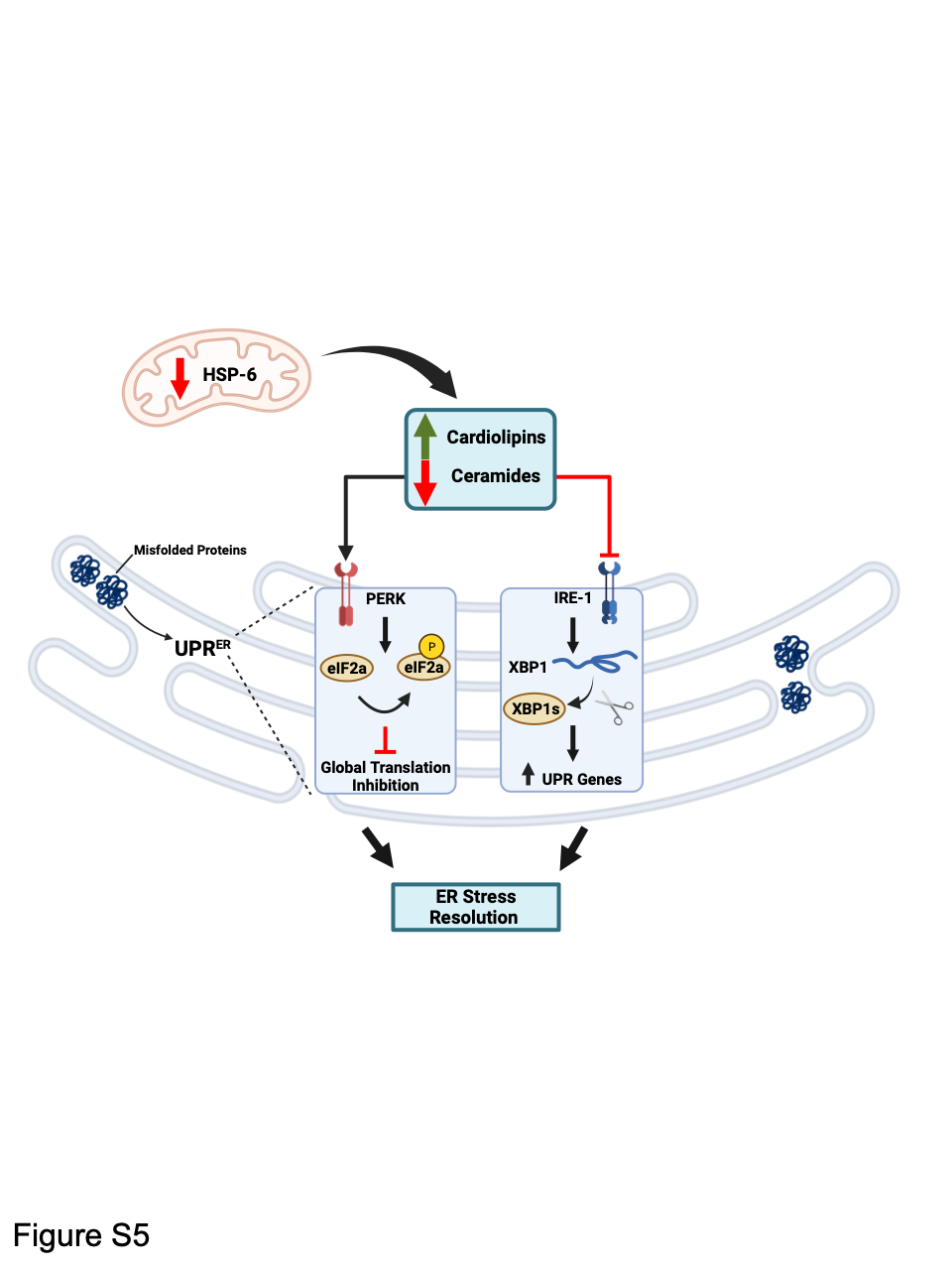

Supplement: S5 Fig — Our findings suggest that the mitochondrial stress response (MCSR), triggered by HSP-6 knockdown, modulates UPRER signaling pathways through alterations in ceramide and cardiolipin levels. As misfolded proteins build up in the ER, the mitochondrial stress response curtails IRE-1 activation and turns-off the expression XBP1 target genes. Simultaneously, it promotes the PERK-dependent eIF2α phosphorylation, leading to a reduction in global protein translation. Consequently, as a newly discovered branch of MCSR, MERSR increases the ER stress threshold and the ER’s protein processing capacity, enhancing overall cellular proteostasis. Created in BioRender. Kim, H. (2025) https://biorender.com/n8x93hs. (TIFF) [file pgen.1011700.s005.tiff]
