# Supplementary material for: Unveiling the intercompartmental signaling axis: Mitochondrial to ER Stress Response (MERSR) and its impact on proteostasis
Source: PLoS Genet. 2025 May 8;21(5):e1011700. doi: 10.1371/journal.pgen.1011700 (PMC12088515; doi:10.1371/journal.pgen.1011700)
Supplement: Attachment — Submitted filename: Response to the Reviewers.docx [file pgen.1011700.s007.docx]

Dear Colleagues,

We sincerely appreciate your time and effort in providing insightful questions and valuable suggestions to improve the findings in this manuscript. We found all the comments extremely helpful in clarifying the proposed mechanism of MERSR.

In response to your feedback, we conducted additional experiments and revised our manuscript accordingly. These revisions have significantly strengthened our findings and further elucidated how this unique mitochondrial stress response regulates ER stress.

Below, we provide a point-by-point response detailing the modifications made. In the revised manuscript, we have highlighted the updated sections for clarity.

We are grateful for your thoughtful and constructive feedback, which has helped refine our work. We hope these revisions address all concerns and that our manuscript is now suitable for publication.

Thank you for your consideration.

Sincerely,

Hyun-Eui Kim

**Reviewers' Comments:**

**Reviewer #1:** This is an interesting study. In previous work, the authors discovered that mitochondria can communicate their proteostasis status to the cytosol, activating cytosolic stress responses that enhance proteostasis in the cytosol. In this manuscript, the authors provide evidence demonstrating that perturbations in mitochondrial proteostasis also influence ER stress responses, a cross-compartmental pathway that the authors call MERSR. Moreover, they identified the ER pathways modulated by alterations in mitochondrial proteostasis (i.e., inhibition of the IRE1 branch and activation of the PERK-eIF2α pathway). They also found that MERSR activation in various disease models ameliorates disease-related protein aggregation and subsequent pathological changes. Together, this study has significant implications for proteostasis and neurodegenerative diseases involving protein aggregation. Nevertheless, I have several comments that I hope the authors will address.

- In the first paragraph of the introduction, the authors briefly mention molecular chaperones and the suppression of global protein synthesis as proteostasis mechanisms. However, they should also include proteolytic systems (especially because ERAD seems to be relevant for the effects reported).

We appreciate the reviewer's valuable insight and feedback. In response, we have incorporated proteolytic systems, including ERAD, into the introduction.

- In the introduction: ‘which compensates for the increase in proteomic stress…’ I’m not sure if ‘proteomic stress’ is correct. This is also used in many other instances throughout the text.

We appreciate the reviewer's feedback on the clarity of terminology. We specifically refer to proteotoxic stress caused by unfolded, misfolded, or aggregated proteins. Accordingly, we have updated the manuscript to consistently use 'proteotoxic stress' or 'protein folding stress' when referring to ER-related unfolded protein stress.

- To further strengthen their conclusions from Figure 2, can the authors test if mtHSP70 RNAi does not further decrease UPRER activation in HYL-1, or HYL-2 or LAGR-1 mutants (any of these genes would be enough, if there available mutant strains for any of them).

We thank the reviewer for suggesting this important experiment. We crossed the SJ4005 (*hsp-4*p::GFP) strain with the *hyl-1* deletion mutant strain (*ok976*) and treated the crossed strain with the indicated RNAi and tunicamycin. As expected, the *hyl-1* deletion mutant significantly reduced UPR^ER^ activation upon tunicamycin treatment. We also tested the *hyl-2* deletion mutant; however, *hyl-2* animals displayed developmental delays, so we conducted our experiments using *hyl-1* animals instead (Figure S2c).

- Can the authors test knockdown levels in Figure 2c to confirm that the knockdown levels for hsp-6 are similar in hsp-6/EV and hsp-6/crls-1?

This is an important point in demonstrating that the knockdown levels are comparable. We included an HSP-6 blot in Figure S2d, which confirms that the knockdown levels were similar.

- In the results sections, many text appear to be more appropriate for the Discussion section. For example: “This suggests that the suppression of UPRER occurs upstream of xbp-1, possibly at a membrane level where IRE1 resides. One possibility is that mtHSP70 knockdown leads to an alteration in membrane fluidity, compromising the dimerization of the UPRER sensor IRE1. This is further corroborated by recent findings that the knockdown of ER-resident proteins alters the ER membrane lipid content, increasing mitochondria and ER contact through increasing membrane order 26. Furthermore, alterations in sphingolipid, including ceramide content within the ER membrane, have been shown to induce UPRER in the absence of aberrant proteostasis 27, providing further evidence that membrane lipid content can directly influence the UPRER signaling pathways”.

We thank the reviewer for the valuable suggestion. We have moved this paragraph to the Discussion section and clarified the Result section accordingly.

- This paragraph in the results section is too long and contains information not directly relevant for the results sections (e.g. discussion about earliest AB models): “Dysfunctional ER stress is a common phenotype in many neurodegenerative diseases, including Alzheimer’s, Huntington’s, and Parkinson’s Disease 35. Early notions of ER stress denoted its function as deleterious 36; however, recent studies have suggested that the response of UPRER in neurodegenerative diseases is highly dynamic 37. Mild UPRER is upregulated during early stages of AD in order to resolve amyloid-β (Aβ) aggregate deposition, however, chronic Aβ burden as a result of advanced AD and constitutive UPRER activation triggers downstream apoptotic pathways, leading to cell death 38. Aβ metabolism is a constant cycle, starting from the synthesis of Amyloid Precursor Protein (APP) in the ER, followed by the export to the Golgi and ultimately to the plasma membrane where it is cleaved by beta-secretase and gamma-secretase to form mature Aβ 39. While an ortholog of APP containing beta-secretase sites is absent in C. elegans, several disease models expressing the disease-causing human Aβ (1-42) peptide have been generated. One of the earliest models of Aβworms was thought to have been able to generate Aβ (1-42) peptides 40, but further research established that the final Aβ product was a truncated version (3-42) due to mis-cleavage of a synthetic signal peptide 41. However, by modifying the artificial signal peptide on the Aβ plasmid, worm strains that produce Aβ (1-42) peptide have now been generated 42.

We agree with the reviewer and have revised the Results section to make it more relevant.

- This sentence appears before the results are explained: “Further evidence suggests that VCP is highly correlated with densely packed polyglutamine aggregates, and upon MERSR activation, a portion of VCP becomes highly associated with loosely packed polyglutamine aggregates”. In the same paragraph, some sentences are more speculative and could fit better into the discussion section. I would suggest to reorganize/rewrite the whole paragraph for clarity.

We agree with the reviewer and have revised the Results section and Discussion section to make it more relevant.

- In Figure S2C, the authors focused on lifespan assays. As a more direct test of physiological consequences in polyQ-expressing worms, the authors could perform motility assays.

We thank the reviewer for the excellent recommendation. We conducted a body bending assay to assess the motility of polyQ-expressing animals with uncontrolled UPR^ER^ activation (Figure S3d). Uncontrolled UPR^ER^ activation, combined with mtHSP70 or ceramide synthase knockdown, further impaired motility, suggesting that MERSR may be detrimental when UPR^ER^ cannot be suppressed.

**Reviewer #2**: In this manuscript the authors expand on their previous findings of a Mitochondrial to Cytosolic Stress Response, and describe a new mitochondrial to ER crosstalk of relevance for C. elegans health. They show that upon ER stress, hsp-6 knockdown impact on both UPRER signaling branches through alteration of lipids (cardiolipin and ceramide) homeostasis: on the one hand hsp-6 RNAi suppress IRE-1 activation of XBP1-regualted genes and on the other it induces PERK-dependent eIF2α phosphorylation, in turn reducing global protein translation. Suppression of these ER regulated signaling axes ultimately by hsp-6 knock down positively impact cellular proteostasis and nematode healthspan in models of proteinopathies. The findings may be thus of interest for a broad audience.

The study is interesting and shed light on a novel intercompartmental signaling axis of relevance for animal’s health. Nonetheless, additional experiments are necessary to substantiate authors’ conclusions before considering the work for publication.

1) What is the effect of hsp-6 RNAi on hsp-4 expression in basal (untreated conditions). Do other ER stressors such as thapsigargin or brefeldin A induce hsp-4 expression in a hsp-6-dependent manner? Also, does overexpression of hsp-6 per se turn on ER stress response?

We appreciate the reviewer to bring up this question. We always have untreated control and they are usually at the basal level. Therefore, we did not include those in the main figure. However, we strongly agree that comparing the reporter induction to its basal level must be shown. Therefore, we included *hsp-4p*::GFP reporter’s untreated controls in the Figure S2c in this revision.

We thank the reviewer’s suggestion on other ER stressors. We treated UPR^ER^ reporter worms with Thapsigargin and Brefeldin A and collected very interesting data.

1) Thapsigargin treatment moderately induced the UPR^ER^ while *hsp-6* knockdown further enhanced UPR^ER^ activation (Figure S1d). This could be due to an imbalance of calcium homeostasis in the ER, cytosol, and mitochondria unlike the Tunicamycin or *cdc-48* RNAi treatment that directly increase protein folding burden in the ER. Therefore, MERSR appears to be independent of calcium-flux signals in the cell.
2) Brefeldin A- induced UPR^ER^ was suppressed by *hsp-6* knockdown (Figure S3c) possibly by reducing global protein translation and protein secretion from ER through MERSR activation.

The observation that *hsp-6* knockdown enhances UPR^ER^ reporter expression during thapsigargin treatment is plausible, as mitochondrial dysfunction exacerbates ER stress due to their interconnected physiology. Additionally, the finding that MERSR does not regulate calcium signaling between the two organelles suggests that its role in modulating UPR^ER^ is independent of calcium dynamics. This highlights how different types of stress can lead to distinct regulatory outcomes for UPR^ER^ and warrants further investigation into how calcium transfer between the mitochondria and ER influences overall stress responses. Moreover, it raises the intriguing question of whether reverse directional signaling from the ER to mitochondria, mediated by calcium balance, contributes to cellular stress adaptation. These findings lay the foundation for exciting follow-up studies.

2) On the other hand, and perhaps more importantly, the first results chapter the author conclude that ER stress is suppressed through the activation of MERSR. To support this conclusion, they should show if ER stress actually activate a MERSR. Namely, what is the effect of ER stressors on hsp-6 expression and more generally on mitochondrial function? Also, mitochondria stress-induced UPR promotes lifespan extension. Does ER-stress also promote lifespan or suppress it? This is also in light of the findings on proteotoxicity.

We thank the reviewer for their insightful comments regarding the directionality of MERSR. To determine whether **ER stress activates MERSR**, we treated the ***hsp-6* reporter strain (SJ4100, *hsp*-6p::GFP)** with **tunicamycin** and found **no induction of *hsp-6* expression** (Figure S2a).

Additionally, we crossed this reporter strain with ***nfyb-1(cu13)* mutant strain**, which induces UPR^ER^, and again detected no increse in **HSP-6 levels** (Figure S2b, lane 1 vs. lane 5). These results align with the previous *C. eleg*ans studies demonstrating that UPR^ER^ does not activate UPR^mt^ or HSR, supporting the notion that UPR^ER^ and UPR^mt^ are distinct and independently regulated pathways (PMID: 23791175).

Interestingly, unlike other stressors that require activation during the developmental stages to induce UPR^mt^, *hsp-6* knockdown in adulthood uniquely facilitates mitochondrial-to-cytosolic (MCSR) and mitochondrial-to-ER (MERSR) communication.

Furthermore, UPR^ER^ **activation has been shown to extend lifespan** through a mechanism distinct from UPR^mt^ **or HSR** (PMID: 23791175). For instance, **neuronal or intestinal *xbp-1s* overexpression** **alleviates proteotoxicity caused by polyglutamine or Aβ aggregates** (PMID: 31315038), similar to the effects of **UPRᵐᵗ activation** (PMID: 27610575) or **HSR activation** (PMID: 28198373). However, the underlying mechanisms of these pathways appear to be **distinct**.

While a detailed investigation of their crosstalk lies beyond the scope of this study, we **agree that exploring potential interconnections** from ER stress to mitochondrial stress responses is an exciting and important direction for future work.

Taken together, our findings indicate that MERSR is activated by mitochondrial stress-not ER stress-and functions to raise the threshold for UPR^ER^ activation. However, under the conditions of severe ectopic overexpression of the proteotoxic proteins that overwhelm ER proteostasis, MERSR activation is insufficient to restore proteostasis and may even exacerbate overall cellular stress.

3) On the same line, at the end of the first results chapter (Figure S1b) the authors show that mrps-5, spg-7 or cco-1 knockdown was unable to reduce UPRER signaling during ER stress. Knockdown of mitochondrial proteins however classically increases hsp-6 expression, which would be expected (opposite to the author speculate) to in fact stimulate hsp-4 expression rather than suppressing it. Why is this not the case?

We appreciate the reviewer's question and the opportunity to clarify this point.

In our study, **MERSR activation** was achieved through ***hsp-6* knockdown**, which induces a **mitochondrial-to-cytosolic stress response (MCSR)** rather than classical **UPRᵐᵗ activation**. Consistent with this, **HSP-6 levels remained low** in this condition (Figure 3e and Figure S2d). Supporting this observation, previous studies have shown that the knockdown of GRP75 (the mammalian ortholog of mtHSP70) protectis against mitochondrial dysfunction in neuronal cells, whereas its overexpression increases sensitivity to oxidative stress via ER-mitochondrial connectivity (PMID: 29367884).

In our experiments, worms were transferred to RNAi plates on day 1 of adulthood to initiate *hsp-6* RNAi. However, RNAi targeting mitochondrial genes such as *cco-1*, *mrps-5*, and *spg-7* must be initiated during the L3 larval stage to efficiently induce *hsp-6* expression and activate UPRᵐᵗ (PMID: 21215371). When these genes are knocked down during adulthood, they fail to robustly activate UPRᵐᵗ or induce *hsp-6* expression.

Moreover, even when UPRᵐᵗ is developmentally activated through these RNAi treatments, it does not induce UPR^ER^ as UPRᵐᵗ and UPR^ER^ operate via distinct regulatory mechanisms (PMID: 23791175).

We hope this explanation helps clarify the differences between UPRᵐᵗ activation timing, MERSR induction, and the interplay between mitochondrial and ER stress responses in our study.

4) In line with previous comment, does alteration of lipids homeostasis (eg knock down of ceramide synthases) affects expression of hsp-6 in basal conditions or upon ER stress? And viceversa, are the two ER stress modulated branches affected by other conditions which impact on hsp-6 expression?

We thank the reviewer for this insightful question.

First, we found that knockdown of ceramide synthases increased HSP-6 expression (Figure S2b). Given that ceramide levels inversely correlate with cardiolipin levels (PMID: 32694663, PMID: 27610574, PMID: 35111371, PMID: 38149844), we speculate that reducing ceramide synthases may elevate cardiolipin levels, which in turn could enhance mitochondrial stress and increase HSP-6 expression.

In contrast, ER stress induced by tunicamycin or by loss of *nfyb-1* did not lead to increased HSP-6 levels (Figure S2a and S2b), suggesting that UPR^ER^ activation does not trigger **UPRᵐᵗ** in our system.

Interestingly, ceramide synthases knockdown strongly suppressed UPR^ER^ (Figure 2a). Moreover, combining *hyl-1* deletion and *hsp-6* knockdown further suppressed UPR^ER^ signaling (Figure S2c, *hyl-1* mutants).

Collectively, these findings suggest that lipid perturbation via ceramide reduction can moderately activate **UPRᵐᵗ** while concurrently suppressing UPR^ER^. However, ER stress does not appear to activate **UPRᵐᵗ**, reinforcing the idea that these pathways are independently regulated in our system.

We hope this response addresses the reviewer’s question and clarifies the relationship between lipid homeostasis and inter-organelle stress signaling.

5) Does mitochondrial stress induced mtUPR (or overexpression of hsp-6) increase the expression of GFP within the coelomocytes or of DAF-28 expression?

We appreciate the reviewer’s continued interest in the relationship between **UPRᵐᵗ** activation and ER function, particularly regarding DAF-28 secretion. To address this, we performed cco*-1* RNAi to moderately induce **UPRᵐᵗ** and examined DAF-28::GFP accumulation within the coelomocytes (Figure S3b). We did not observe any significant changes in basal levels of protein secretion or in the expression of the secreted protein.

These results are consistent with our previous findings and further support the conclusion that classical **UPRᵐᵗ** activation does not ubtantially impact ER function in this context.

We hope this clarifies our observations and appreciate the opportunity to expand on this point.

6) Previous work has clearly demonstrated that induction of the mtUPR has beneficial effects against Abeta induced proteotoxicity. Nature. 2017 Dec 14;552(7684):187-193. doi: 10.1038/nature25143. How do the authors reconcile their findings with previously published work? Does hsp-6 knockdown impact on wild-type animals’ lifespan in basal conditions or upon ER-induced stressors?

We appreciate the reviewer’s reference to previous work demonstrating the beneficial effects of **UPRᵐᵗ** induction on Aβ-induced proteotoxicity. However, our study focuses specifically on the effects of *hsp-6* knockdown, which differs mechanistically from classical **UPRᵐᵗ** activation (PMID: 21215371, PMID: 27610574).Canonical **UPRᵐᵗ** activation, as studied in the referenced work, is typically induced during development and has been associated with protection against proteotoxicic stress. In contrast, *hsp-6* knockdown in our study is applied during adulthood, triggering MCSR (PMID: 27610574) and MERSR, which exhibit distinct regulatory features from evelopmental **UPRᵐᵗ**.

We observied that *hsp-6* knockdown moderately extends the lifespan in wild-type animals under basal conditions (Figure S4a). However, in the context of ER stress conditions (e.g., *nfyb-1* mutant animals), *hsp-6* knockdown does not extend lifespan, suggesting that MERSR does not provide the same protective effect as classical **UPRᵐᵗ**.

Moreover, we found that *hsp-6* knockdown modestly improved paralysis phenotyps in both neuronal and muscle Aβ-expressing *c. elegans* models (Figure 4a and S4b). (The same muscle Aβ- expressing worms were utilized in the referenced work).

The protective effects of developmental **UPRᵐᵗ** activation in neurodegenerative disease models may result from early-life preconditioning or epigenetic regulation (PMID: 27610575, PMID: 30057120, PMID: 27133168). In contrast, our findings highlight a distinct role for mitochondrial stress responses induced in adulthood particularly in terminally differentiated tissues.

Finally, the observation that *hsp-6* knockdown does not extend lifespan under ER stress conditions further supports the distinction between MERSR and classical **UPRᵐᵗ.**

We hope this clarifies the relationship between our findings and prior literature, and we appreciate the opportunity to explain this distinction in more detail.

7) Does amelioration of the proteotoxicity also rely on different components of the ER-stress response branches ie suppress IRE-1 and activation of XBP1-regualted genes and PERK-dependent eIF2α pathway?

We appreciate the reviewer’s thoughtful question regarding the involvement of pecific ER stress response branches in the amelioration of proteotoxicity.

Our data indicate that MERSR primarily acts through the PERK/eIF2α pathway. Specifically, *hsp-6* knockdown enhances eIF2α phosphorylation (Figure 3e) and when *pek-1* (PERK) is knocked down in combination with *hsp-6*, the suppression of UPR^ER^ was no longer observed (Figure S3a). These findings strongly suggest that MERSR activation is PERK-dependent.

To evaluate the role of the IRE1/XBP1 branch, we performed *xbp-1* knockdown in animals expressing Aβ(1-42) or polyglutamine. In both models, *xbp-1* knockdown exacerbated proteotoxicity (Figure S3d), inditating that the IRE1/XBP1 pathway contributes to proteostasis maintenance, particularly when MERSR is inactive or impaired.

Together, these results suggest that MERSR promotes proteostasis primarily through PERK/eIF2α signaling, while the IRE1/XBP1 axis may serve as a complementary or compensatory pathway under conditions when MERSR is not engaged.

We appreciate the opportunity to clarify these mechanistic insights and hope this response addresses the reviewer’s inquiry.

Minor comments
- In the introduction the authors refer to own previous work indicating intercompartmental crosstalk. They should cite the related studies.

Thank you for the pointing out the missing references. We have updated them.

- It is not clear why the authors in the text write mtHSP70 while in fact they are working and referring to it in the figure to C. elegans hsp-6 RNAi. Should keep it consist for simplicity.

We appreciate the reviewer’s attention to clarity and consistency in terminology. In our manuscript, we refer to mtHSP70 to highlight its conserved role across species, as it is functional equivalent of mammalian mtHSP70 (also known as GRP75, mortalin, or HSPA9). However, we fully acknowledge that in *C. elegans*, the specific homolog is *hsp-6*, and it is important to distinguish this in the context of our experimental system.

To avoid confusion and improve consistency, we have revised the manuscript to refer to hsp*-6* RNAi when describing experiments conducted in *C. elegans.* We will reserve the use of “mtHSP70” for broader discussions where cross-species comparisons are relevant.

We appreciate the reviewer for this helpful suggestion, which has improved the clarity of the manuscript.

- The authors report (Figure S1a) that dev-1RNAi also suppresses ER stress response induction. Why this is also not the case for atfs-1?

This is a very important question, and while we do not yet have a definitive answer, our data suggest that MERSR operates independently of ATFS-1*.* Several ATFS-1 independent pathways have been identified that regulate mitochondrial stress responses and may account for the observed suppression of UPR^ER^ by *dve-1* RNAi but not *atfs-1* RNAi

1) DVE-1depedent stress response: Some mitochondrial stress responses are regulated via the transcription factor DVE-1, which does not require ATFS-1 for nuclear translocation (PMID: 39423131).
2) Mitochondrial integrated stress response (ISR): Mitochondrial stress can also lead to eIF2α phosphorylation, and ISR activation, which functions independently of ATFS-1 (PMID: 39608727).
3) Mitophagy and autophagy: Certain mitochondrial stress responses are mediated through mitophagy and autophagy pathways that do not requireATFS-1(PMID: 38340717).

These ARFS-1-independent mechanisms may allow mitochondrial stress to influence ER stress responses through alternative signaling routes, including MERSR.

We appreciate the reviewer raising this important mechanistic distinction, and we believe it represents an exciting direction for future research.

- In figure 4 the authors use opposite colors for EV and hsp-6 legends, which is somewhat confusing.

We appreciate the reviewer’s attention to figure clarity. We understand how the use of opposite colors for EV and *hsp-6* in Figure 4 may have caused confusion. In response, we have revised the figure to ensure that the color scheme is consistent across all panels.

Thank you for bringing this to our attention. This adjustment improves the overall readability of the figure.

- In the last results’ chapter, the authors use a strain which they claim to be neuronal RNAi-sensitive. Yet this has been reportedly shown to display RNAi sensitivity also in other tissues. Results should be therefore discussed accordingly.

We appreciate the reviewer’s comment regarding the RNAi sensitivity of the TU3401 strain. It is indeed known that TU3401 can exhibit RNAi effects in non-neuronal tissues at later stages of adulthood, as the system may become “leaky” over time. A more tightly controlled, neuron-specific RNAi strain has been developed recently (PMID: 31827090), which may offer greater specificity in future studies. Nonetheless, **TU3401 still enables neuronal knockdown** of target genes**.** To ensure transparency, we have added a cautionary note in the figure legends acknowledging the potential for off-target RNAi effects in non-neuronal tissues at later stages.

We thank the reviewer for this important suggestion, which has improved the rigor and clarity of our manuscript.

- MERC and ceramides are involved in apoptosis. It would be intersting to know whether any of the effects modulated by MERSR are mediated by apoptotic-regulatory genes?

We appreciate the reviewer’s insightful question regarding the potential involvement of apoptotic regulatory genes in MERSR-mediated effects, especially given the known roles of mitochondria–ER contacts (MERC) and ceramides in apoptosis.

While our current study did not directly assess the involvement of apoptosis-related genes, we agree that investigating how MERC remodeling or ceramide signaling might influence apoptotic pathways in the context of MERSR is an important and intriguing future direction. It is also worth noting that *C. elegans* apoptosis does not depend on cytochrome c release, and as such, the mechanistic relevance of MERC in apoptosis may be more fully captured in mammalian systems.

We will certainly take this into consideration as we expand our studies into mammalian models, where we aim to explore more deeply the links between MERSR, apoptosis regulation, and broader MERC-associated functions.

**Have all data underlying the figures and results presented in the manuscript been provided?**
Large-scale datasets should be made available via a public repository as described in the *PLOS Genetics* [data availability policy](https://nam04.safelinks.protection.outlook.com/?url=http%3A%2F%2Fc05y1x9s.r.us-east-2.awstrack.me%2FL0%2Fhttp%3A%252F%252Fjournals.plos.org%252Fplosgenetics%252Fs%252Fdata-availability%2F2%2F010f0192b5cac250-f433ff64-a89d-4a7c-b67c-e8ab78eeab2b-000000%2FJS0rKkf-TSNxOaXJAdfAPKHhqEk%3D182&data=05%7C02%7Chyun-eui.kim%40uth.tmc.edu%7C283f7f58328e4350e76908dcf2d3387d%7C7b326d2441ad4f57bc6089e4a6ac721b%7C0%7C0%7C638652236239735789%7CUnknown%7CTWFpbGZsb3d8eyJWIjoiMC4wLjAwMDAiLCJQIjoiV2luMzIiLCJBTiI6Ik1haWwiLCJXVCI6Mn0%3D%7C0%7C%7C%7C&sdata=qaSZf2Ops4aHz%2FVUchAQMjgPFS4FKaZ%2BOC%2B3DhfXl%2FI%3D&reserved=0), and numerical data that underlies graphs or summary statistics should be provided in spreadsheet form as supporting information.

Reviewer #1: Yes

Reviewer #2: Yes
